# University of the State of New York1Published by permission of the Director of Examinations.

**Published:** 1895-04

**Authors:** 


### UNIVERSITY OF THE STATE OF NEW YORK.[1]

REGENTS' OFFICE, ALBANY, N. Y.

Extract from 1894 Report of the Examination Department. (Advance Sheets.)

[MEDICAL.]

AN ANALYSIS of the results of the licensing examinations since September 1, 1891, shows:

That 8.9 per cent. of the old school[2] candidates were rejected in 1892, 7.4 per cent in 1893 and 20.2 per cent. in 1894; that 25 per cent. of the homeopathic candidates were rejected in 1892, 9.5 per cent. in 1893, and 13.7 per cent. in 1894; that 50 per cent. of the eclectic candidates were rejected in 1892, 28.5 per cent. in 1893 and 50 per cent. in 1894.

During the three years 56, 267 and 390 old school candidates have been examined, 8, 21 and 51 homeopaths and 4, 7 and 4 eclectics, making a total of 808, of which number 121, or 15 per cent., were rejected.

This result is remarkable when we consider that admission to the licensing examinations, presupposes the preliminary education required by statute and graduation with the degree of bachelor or doctor of medicine from a registered medical school.

---

1. Published by permission of the Director of Examinations.

2. This term is as it appears in the report. We are not responsible for its use.—EDITOR.

It speaks well for New York State medical schools that of the 445 candidates examined during the year ending July 31, 1894, 291 were graduates of New York medical schools and 154 of schools of other states and countries, and that of the eighty-eight candidates rejected at these examinations forty-two were graduates of New York medical schools and forty-six of schools of other states and countries. The detailed report which follows gives the record for each institution.

The work of the older practitioners compared favorably in 1893 with that of the recent graduates:

| | | | | | |
|---|---|---|---|---|---|
| Of those examined on graduation....... | 8.2 | per cent. | were | unsuccessful |
| " " " after one year's practice............................ | 6.2 | " | " | " | " |
| Of those examined after two years' practice.............................. | 16.6 | " | " | " | " |
| Of those examined after not less than three or more than thirty-three years' practice.................. | 10.8 | " | " | " | " |

In 1894, the statistics are not so favorable to the older practitioners, being respectively 13, 23, 14 and 44 per cent. It will be noted that candidates two years out of college did far better than those examined after one year and about as well as recent graduates. Of the thirty-one rejected candidates who had practised for at least three years, seven had practised three years, six four years, three five years, eight from six to nine years and only seven more than nine years.

Failures for the year in the several topics were as follows: Anatomy, 12; physiology and hygiene, 12; chemistry, 18; surgery, 17; obstetrics, 44; pathology and diagnosis, 33; therapeutics, practice and materia medica, 15.

It is remarkable that old practitioners do better in anatomy and in chemistry than in obstetrics or pathology and diagnosis. These statistics show at all events that in the only subject for which some have demanded two standards, the old practitioners do not need special dispensations.

Dr. Perry H. Millard tells us that in the decade from 1880 to 1890 medical schools in the United States matriculated 115,355 students and graduated 40,996, or an average of more than 4,000 annually, in his opinion twice as many as the requirements of the people demand.

As New York is the acknowledged center of medical education in the United States, we should naturally expect the number of physicians graduated annually by her medical schools to be greater in proportion to her inhabitants than that in other states. Under former laws between 600 and 800 physicians were graduated yearly by New York medical schools.[1] On the basis of population alone the quota of physicians for New York should not exceed 400. Though under the present law the total number licensed annually will be about 400, (365 from January 1, 1895,) yet as the New York schools furnish physicians for other states. they will probably continue to graduate a larger number than apply for license (536 from January 1, 1894, to January 1, 1895).

The record of the past year proves conclusively the statements made in the 1893 report that the new law, while an effective barrier to the ingress of the imcompetent, has also operated to raise the standard of preliminary education and to improve terms of study and methods of teaching in the medical schools. The law is now quite satisfactory with the exception of the preliminary requirement, which is still far too low, and which we hope will be advanced so that with the class matriculating in 1897, at least a full high school course will be required. The second defect is in the section relating to penalties and their collection. A bill to correct this defect passed both houses of the 1894 legislature, but failed to receive the approval of the governor.

Perhaps the strongest argument in behalf of adequate medical legislation is found in the statistics based on the proportion of physicians to the number of inhabitants in a few of the European countries, as compared with the number in the Uuited States. These statistics are given as follows in the admirable paper of Dr. Millard, read in 1892 before the American Academy of Medicine, at Detroit, Michigan :

RATIO OF PHYSICIANS TO POPULATION.

| | |
|---|---|
| Sweden | 1 to 7,000 |
| Italy | 1 to 3,500 |
| Germany | 1 to 3,000 |
| Austro-Hungary | 1 to 2,400 |
| France | 1 to 2,000 |
| United States | 1 to 600 |

1. In 1887, 610 ; 1888, 632 ; 1889, 611 ; 1890, 739 ; 1891, 790 ; 1892, 661 ; 1893, 620 ; 1894, 553.

Dr. William Pepper, in an appendix to his instructive addresses on Higher Medical Education, published in 1894, revises some of these figures as follows :

| | |
|---|---|
| Italy | 1 to 3,536 |
| Germany | 1 to 3,038 |
| Austro-Hungary | 1 to 3,857 |
| France | 1 to 2,666 |

He also publishes a still more significant table, from which we reprint a few statistics :

| | Population. | No. of Physicians. | No. of Medical Schools. | Proportion of Schools to Population. |
|---|---|---|---|---|
| Sweden | 4,802,751 | ...... | 3 | 1 to 1,600,917 |
| Italy | 30,347,291 | 8,580 | 21 | 1 to 1,445,109 |
| Germany | 49,428,470 | 16,270[1] | 20 | 1 to 2,471,923 |
| Great Britain | 37,740,285 | 22,105 | 16[2] | 1 to 2,358,767 |
| Austro-Hungary | 41,231,342 | 10,690 | 8 | 1 to 5,153,917 |
| France | 38,343,139 | 16,593 | 7 | 1 to 5,477,591 |
| United States | 62,622,250 | 100,000 | 140 | 1 to 440,151 |

If we compare the proportion of schools to population in the United States as given in the census of 1890, we find the ratio 1 to 428,418, in New York ; 1 to 559,736, in Massachusetts ; 1 to 193,-279, in Ohio ; 1 to 425,190, in Illinois ; 1 to 876,335, in Pennsylvania, and elsewhere from 1 to 57,598 in the District of Columbia to 1 to 2,235,525 in Texas, with only six states in which the ratio is more than 1 to 1,000,000.

There are more medical schools in the United States alone than in countries whose total population is six times as great, and yet less than one dozen of these medical schools in the United States are endowed.

Under these conditions we must expect ridiculously low requirements both for matriculation and for graduation where the public is not protected by statutes which establish minima standards.

Of the 140 medical schools in the United States there are, we believe, only fourteen[3] which absolutely require a four-year course

1. Including officers of health.

2. Not including hospitals in which instruction is given, diplomas for which office were abolished December 30, 1892.

3. *New York*—College of Physicians and Surgeons, Medical College and Hospital for Women, Homeopathic Medical College and Hospital, Woman's Medical College of the New York Infirmary ; *Pennsylvania*—University of Pennsylvania, Woman's Medical College of Philadelphia ; *Massachusetts*—Harvard University Medical School, Boston University School of Medicine ; *Michigan*—Department of Medicine and Surgery, University of Michigan, Homeopathic Medical College, University of Michigan ; *Illinois*—Northwestern University Medical School ; *Tennessee*—Maharry Medical Department of Central Tennessee College ; *Maryland*—Johns Hopkins Medical School ; *North Carolina*—Leonard Medical School.

of medical lectures. Nearly 100 schools announce that they graduate on three terms of lectures, and about twenty-five on two terms, the length of the term varying from five to nine months.

Most foreign countries, including some regarded by us as only semi-civilized, require a four, five or six year medical course after completion of rigorous preliminary requirements.

A six year medical course is required in Guatemala, Turkey, Egypt, Argentine Republic, Brazil, Chili, Italy, Netherlands (average time seven years), Norway (six to eight years), Sweden (usual duration of medical studies ten years), Uruguay, Venezuela; a five year medical course in Great Britain, Portugal, Russia, Roumania, Mexico, Switzerland, Peru (for B. D.—seven years for license); a four year medical course in Austro-Hungary, France (frequently more than five years), Germany (for M. D., except at Erlangen, which is three years—for license, six years), Hayti, Japan, Spain (for M. D.—six years for license).

Matriculation requirements in most foreign countries are still more exacting as compared with our standards. In Germany the matriculant must have passed the *Abiturientenexamen* after completing the course of nine years in a *Gymnasium*. This examination presupposes as much work as is usually covered at the close of the sophomore year in a registered American college. Pharmacists must have passed the examination at the close of six years in a *Gymnasium or Realschule* (*Zeugniss zum einj. Dienste*), dentists and veterinary surgeons, that at the close of seven years (*Zeugniss der Reife fuer eine Gymn. oder einer Realschule*).

In Austro-Hungary, Russia and Switzerland, the requirements for matriculation to medical study are on about the same plane. In France the degree of *Bachelier és lettres* (equivalent to graduation from a full high school classical course), and additional examination in physics, chemistry and the natural sciences are required. A similar standard is maintained in Italy (*Licenza liceale*), Spain and Cuba (*Bachillerato* and a supplementary examination). In England the minimum test, fixed by the program of the British medical council, like our own is comparatively low, though many English students either have passed a matriculation examination in arts or hold the B. A. degree. In the Canadian provinces also the varying requirements do not represent in any case the completion of a registered high school course and are not as high as Germany exacts from students of pharmacy, dentistry or veterinary medicine.

